# Cotton Recruits Soil‐Derived *Delftia tsuruhatensis* to Suppress Aphid Detoxification Via Salicylic Acid‐Mediated Defense

**DOI:** 10.1002/advs.75321

**Published:** 2026-04-20

**Authors:** Hui Xue, Fengchao Diao, Qiqing Yu, Xiangzhen Zhu, Tingfang Zhong, Li Wang, Kaixin Zhang, Dongyang Li, Jichao Ji, Junyu Luo, Jinjie Cui, Xueke Gao

**Affiliations:** ^1^ Research Base of Zhengzhou University, State Key Laboratory of Cotton Bio‐Breeding and Integrated Utilization Institute of Cotton Research Chinese Academy of Agricultural Sciences Anyang Henan China; ^2^ College of Plant Science and Technology Huazhong Agricultural University Wuhan Hubei China; ^3^ State Key Laboratory of Cotton Bio‐Breeding and Integrated Utilization, School of Agricultural Sciences Zhengzhou University Zhengzhou Henan China

**Keywords:** aphids, bacterial recruitment, cotton, detoxification metabolism, salicylic acid

## Abstract

The synergistic defense between microorganisms and plants holds significant ecological importance in resisting pest attacks. Herbivorous insects pose a significant threat to global agriculture. While plants deploy defense hormones like jasmonic acid (JA) and salicylic acid (SA), the role of beneficial microbes in enhancing these defenses remains underexplored. This study unveils a novel tripartite interaction in which cotton actively mobilizes the soil‐derived bacterium *Delftia tsuruhatensis* to roots and leaves upon aphid attack, reshaping its microbiome for defense. Following aphid infestation, the abundance of *D. tsuruhatensis* was increased significantly in both roots and leaves. Inoculation of cotton with *D. tsuruhatensis* significantly reduced aphid fitness, inhibiting phloem feeding, decreasing survival, prolonging nymphal development, and impairing reproduction. Mechanistically, we found that *D. tsuruhatensis* activates the host plant's SA signaling pathway. This plant‐mediated response, in turn, effectively suppresses the expression of a key aphid detoxification gene, *UGT2B17* (*UDP‐glucosyltransferase 2B17*), compromising the insect's ability to metabolize plant defenses. Furthermore, the combination of *D. tsuruhatensis* and RNAi‐mediated silencing of *UGT2B17* synergistically enhanced aphid mortality. Our results elucidate a sophisticated defense strategy wherein the host plant leverages a beneficial microbe to augment its innate immunity through cross‐kingdom gene regulation, ultimately disarming the pest's detoxification system.

## Graphical abstract

This study systematically reveals a complex interactive network involving plants, microbes, and insects, elucidating the ecological and molecular mechanisms by which cotton enhances its resistance to aphids through the active recruitment of the beneficial soil bacterium *Delftia tsuruhatensis*.

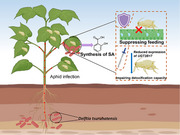

## 1 Introduction

Herbivorous insects pose a major threat to plant growth and cause substantial annual losses in global crop production [1]. In response to pest attacks, plants have evolved robust defense strategies, including the establishment of physical barriers, production of toxic metabolites, and activation of hormone‐mediated responses [2]. Phytohormones play central roles in regulating plant defense mechanisms. Among them, jasmonic acid (JA) and salicylic acid (SA) are the primary defense‐related hormones, while others, such as ethylene (ET), abscisic acid (ABA), auxins, gibberellins (GAs), cytokinins (CKs), and brassinosteroids (BRs) are also involved in defense responses [3, 4]. The antagonistic or synergistic interactions among these hormones constitute a complex signaling network that underlies plant immunity under stress conditions [4].

Although plant defense mechanisms have been extensively characterized, how these defenses function in the context of plant interactions with other organisms—such as beneficial microbes—remains an area requiring further investigation. The plant microbiota, often referred to as the plant's “second genome”, provides numerous benefits to the host, including enhanced growth, enhanced nutrient acquisition, improved stress tolerance, and resistance to pathogens [5, 6]. In particular, microbe‐mediated plant defense against insects has emerged as a compelling research topic [7, 8] and offers promising potential for environmentally friendly pest management. One study demonstrated that plants under insect herbivory can recruit insecticidal *Pseudomonas* species on the phyllosphere—the leaf surface microbiome—thereby enhancing resistance against further insect attacks [7]. Additionally, plant symbionts often modulate phytohormone pathways involved in defense responses, thereby providing the host with additional defensive capabilities that facilitate successful colonization and symbiosis [9, 10, 11]. These mechanisms frequently involve the upregulation of SA and JA pathways, ET signaling, or the induction of protective proteins [12]. Therefore, elucidating the synergistic relationships between plant defense responses and symbiotic microorganisms is of considerable ecological importance for developing efficient and sustainable pest control strategies.

Aphids (Hemiptera: Aphididae) are among the most detrimental pests in global agricultural production [13]. Their rapid life cycles, diverse reproductive strategies, and broad environmental adaptability amplify their destructive impact on crop yields [14]. Aphid feeding can cause plant deformation, wilting, growth retardation, transmission of plant diseases, and severe yield losses [15, 16]. Throughout prolonged co‐evolution, aphids and their host plants have developed a complex antagonistic relationship, continuously influencing each other's traits and adaptability. This ongoing arms race has driven the evolution of highly specialized adaptive mechanisms [17]. In response to plant immunity, aphids have evolved various strategies to circumvent host defenses, primarily through the production of detoxifying enzymes and behavioral adaptations in feeding [18, 19]. Moreover, the synergistic role of plant‐associated microbes in enhancing resistance presents a fascinating scientific question with practical implications. Thus, future pest management strategies should not only rely on chemical control but also exploit plant–microbe interactions to develop effective biological control measures as a sustainable direction for green agriculture.

In this study, we investigated the globally significant pest *Aphis gossypii* and its host plant, cotton, unveiling a novel defense mechanism for the first time within the cotton–aphid–microbe interaction system. Specifically, insect infestation led to a specific enrichment of the beneficial bacterial genus *Delftia*. Cotton leaves treated with *Delftia tsuruhatensis*, isolated from the leaves, exhibited a significant reduction in aphid fitness. Mechanistically, this bacterium enhances cotton resistance by activating the SA signaling pathway in plants and suppressing the expression of the aphid detoxification enzyme gene *UGT2B17* (*UDP‐glucosyltransferase 2B17*), thereby limiting aphid infestation and population expansion. These findings not only expand the theoretical framework of plant–insect–microbe interactions, providing new ecological and molecular insights into insect population regulation, but also establish a solid foundation for developing probiotic‐based green strategies for aphid control—such as those employing *D. tsuruhatensis*. This work holds significant potential for advancing sustainable agricultural practices.

## 2 Experimental Section

### 2.1 Insects and Plants

The cotton aphids (*A. gossypii*) were originally collected from experimental fields at the Cotton Research Institute of the Chinese Academy of Agricultural Sciences in 2018. The aphids were maintained through long‐term rearing in an artificial climate‐controlled chamber set at 26°C ± 1°C, 65% ± 5% relative humidity, and a 14‐h light/10‐h dark photoperiod. The upland cotton variety (*Gossypium hirsutum* cv. ICR49) was cultivated under greenhouse conditions with consistent parameters: temperature maintained at 26°C ± 1°C, relative humidity at 65% ± 5%, and a photoperiod of 14 h light and 10 h darkness.

### 2.2 Microbiome Sample Collection, DNA Extraction, and 16S rRNA Sequencing

At the sixth day of the cotyledon stage of cotton plants, ten one‐day‐old adult cotton aphids were transferred onto each plant to establish the infestation group, while control plants remained untreated. Upon the emergence of the first true leaf, the aphids were removed, and both cotyledons and roots were collected. The samples were surface‐sterilized with 75% ethanol in a laminar flow cabinet, followed by thorough rinsing with sterile water to eliminate any residual ethanol. Cotyledons and roots from twenty plants were pooled as one biological replicate for leaf and root tissues, respectively, yielding a total of six replicates. All samples were immediately flash‐frozen in liquid nitrogen and subsequently stored at −80°C.

Genomic DNA was extracted from the samples using the TIANamp Genomic DNA Kit (TIANGEN, China) according to the manufacturer's instructions. The quality of the DNA was assessed by 1% agarose gel electrophoresis, and concentration was determined using a NanoDrop 2000 spectrophotometer (Thermo Scientific, United States). Amplification of the bacterial 16S rRNA gene V5–V7 hypervariable regions was performed with the primers 799F and 1193R under the following PCR conditions: initial denaturation at 95°C for 3 min; 27 cycles of denaturation at 95°C for 30 s, annealing at 55°C for 30 s, and extension at 72°C for 30 s; followed by a final extension at 72°C for 5 min. The PCR products were excised from 2% agarose gels, purified using a PCR Clean‐Up Kit (YuHua, Shanghai, China) according to the manufacturer's protocol, and quantified with a Qubit 4.0 fluorometer (Thermo Fisher Scientific, USA). The purified amplicons were used for library construction with the NEXTFLEX Rapid DNA‐Seq Kit (Revvity, United States). Sequencing was performed with the Illumina MiSeq PE300 platform (Majorbio Bio‐Pharm Technology Co., Ltd., Shanghai, China). All raw data were uploaded to the NCBI SRA database (BioProject accession number: PRJNA1321557).

### 2.3 16S rRNA Sequence Data Processing

Raw paired‐end sequencing reads were processed using fastp [20] for quality control and FLASH [21] for assembly according to the following steps: (1) Trimming of bases with a quality score below 20 at the read tails; removal of reads shorter than 50 bp; and removal of reads containing ambiguous nucleotides (N); (2) Merging of paired‐end reads into single sequences based on overlap relationships, with a minimum overlap length of 10 bp; (3) Filtering of assembled sequences with a maximum mismatch ratio of 0.2 within the overlap region; (4) Demultiplexing of sequences according to barcodes and primers at both ends with direction adjustment. No mismatches were allowed in barcodes, and up to 2 mismatches were permitted in primers.

Processed and assembled sequences were subjected to denoising using the DADA2 plugin [22] in QIIME 2 [23] with default parameters. The resulting sequences were referred to as amplicon sequence variants (ASVs). Sequences annotated as chloroplasts or mitochondria were removed from all samples. To minimize the impact of sequencing depth on subsequent alpha and beta diversity analyses, all samples were rarefied to 20 000 sequences per sample. After rarefaction, the average sequence coverage (Good's coverage) remained at 99.09%. Taxonomic assignment of ASVs was performed using the naive Bayes classifier in QIIME 2 based on the SILVA 16S rRNA gene database (v138).

All data analyses were conducted on the Majorbio Cloud platform [24]. Alpha diversity was evaluated using the ACE (Abundance‐based Coverage Estimator) index calculated in mothur [25], and group differences were assessed using Student's *t*‐test, with significance levels denoted as ^**^ for 0.001< *p* < 0.01. Similarity in microbial community structure between samples was visualized using principal coordinate analysis (PCoA) based on the *abund jaccard* distance. Permutational multivariate analysis of variance (PERMANOVA) was applied to test for significant differences in microbial community structure between groups, with *p* < 0.05 considered statistically significant. Differential abundance of bacterial genera was analyzed using Student's *t*‐test, with ^*^ indicating 0.01< *p* < 0.05 and ^**^ indicating 0.001< *p* < 0.01. Linear discriminant analysis (LDA) was used to measure the magnitude of the effect of species on the observed differences, and the resulting LDA scores indicated that the abundance of the representative species plays a key role in driving these differences.

### 2.4 Isolation and Identification of Bacteria in Cotton

Bacteria were isolated from both cotyledons and roots of cotton plants at the four‐leaf stage. Leaf samples were surface‐sterilized by immersion in 75% ethanol for 40 s, followed by treatment with 2.5% sodium hypochlorite for 5 min, and finally rinsed thoroughly with sterile water. The sterilized leaves were homogenized in sterile PBS buffer, and the resulting supernatant was spread onto both NA (Nutrient Agar) and R2A agar media. Incubation was carried out at two temperatures (28°C and 37°C) for 24 h. Individual colonies were selected using a sterile loop and inoculated into conical flasks containing liquid NA or R2A medium, followed by shaking at 120 rpm for further cultivation. Bacterial sequencing was performed by Sangon Biotech Co., Ltd. (Shanghai, China). Species identification was conducted by comparing the obtained sequences against the NCBI database using the blastn algorithm. Bacterial suspensions were mixed with sterilized 50% (v/v) glycerol at a final glycerol concentration of 25%, and stored at −80°C under designated identifiers. Details of the bacterial isolates from cotton leaves are provided in Table S1. *D. tsuruhatensis* investigated in this study was isolated using R2A medium.

### 2.5 Identification of the Source of *D. Tsuruhatensis*

Three distinct growth environments were established for cotton plants: (1) plants grown under standard laboratory conditions without aphid infestation; (2) plants grown under standard laboratory conditions but subjected to aphid infestation; and (3) plants cultivated under sterile conditions in closed containers using 1/2 MS medium (Solarbio, Beijing, China, Cat: M8525) without soil contact. “Standard laboratory conditions” refers to soil cultivation under non‐sterile environments. The 1/2 MS medium was prepared at a concentration of 4% (w/v) according to the manufacturer's instructions and poured into sterilized glass containers. Surface‐sterilized cotton seeds were germinated and grown until the four‐leaf stage in separate containers. From each of the three environments, root samples, leaf samples, and soil (where applicable) were collected, with each category comprising 60 individual replicates.

Quantification of *D. tsuruhatensis* copy number: DNA from soil samples was extracted using the FastDNA Spin Kit for Soil (MP Biomedicals, USA), while DNA from cotton tissues was isolated with the TIANamp Genomic DNA Kit (TIANGEN, China), each following the manufacturer's protocols. Plasmid DNA extracted from a pure culture of *D. tsuruhatensis* using the TIANprep Mini Plasmid Kit (TIANGEN, China) was serially diluted and used as a standard curve for absolute quantification. Quantitative PCR (qPCR) was performed on the StepOnePlusTM Real‐Time PCR System (Applied Biosystems, Waltham, MA, USA) using TransStart Top Green qPCR SuperMix (TransGen Biotech, China) validated primers in 10 µL reaction volumes. Thermal cycling parameters included: 95°C for 3 min; 40 cycles of 95°C for 5 s and 60°C for 30 s. Primer information is provided in Table S2.

Semi‐quantitative detection of *D. tsuruhatensis*: DNA samples from each category described above were pooled and subjected to PCR amplification using PrimerSTAR Max DNA Polymerase (Takara, Japan) under the following cycling conditions: initial denaturation at 98°C for 5 min; 40 cycles of denaturation at 98°C for 20 s, annealing at 55°C for 30 s, and extension at 72°C for 30 s; followed by a final extension at 72°C for 5 min. The reaction mixture was prepared according to the manufacturer's recommendations. PCR products were analyzed by electrophoresis on a 1% agarose gel. Primer sequences are listed in Table S2.

### 2.6 Preliminary Evaluation of Insecticidal Activity in Bacteria of Cotton

Forty‐one isolated bacterial strains were individually cultured in their appropriate liquid media (NA or R2A) until the OD_600_ reached 0.8. Bacterial suspensions were evenly sprayed onto detached cotton cotyledons with 500 µL per leaf. One‐day‐old aphids were placed on the treated leaves, and the leaves were replaced every 24 h with freshly sprayed cotyledons. Control groups were treated with sterile water, NA medium, or R2A medium, respectively, following the same application procedure. The developmental stages of the aphids were recorded at 12‐h intervals. The weight of fourth‐instar nymphs was measured using an XS205DU analytical balance (Mettler Toledo, Greifensee, Switzerland). Once the aphids began reproducing, the number of offspring produced and individual mortality were recorded every 24 h. The bioassay included 60 aphids per treatment.

### 2.7 Transcriptome Sample Collection, RNA Extraction, and Sequencing

Based on the survival curve of cotton aphids treated with *D. tsuruhatensis*, two time points were selected for sampling: day 8 (point of sharp decline in survival) and day 4 (mid‐point). *D. tsuruhatensis* was cultured in R2A liquid medium until OD_600_ reached 0.8. To eliminate potential effects of the medium itself, the bacterial cells were pelleted by centrifugation at 3000 rpm, washed, and resuspended in an equal volume of sterile water. The resuspended bacterial solution was evenly sprayed onto detached cotton cotyledons with 500 µL per leaf, followed by inoculation with one‐day‐old aphids. Treated leaves were replaced every 24 h, with fresh bacterial suspension reapplied each time. Control groups received sterile water instead. Aphids were collected on days 4 and 8. All samples were immediately flash‐frozen in liquid nitrogen and stored at −80°C.

Total RNA was isolated from samples using TRIzol reagent (Thermo Fisher Scientific, Carlsbad, CA, USA) according to the manufacturer's protocol. RNA integrity was verified through 1% agarose gel electrophoresis, while concentration and purity were determined using a NanoDrop 2000 spectrophotometer (Thermo Fisher Scientific, Waltham, MA, USA). Library preparation and sequencing were performed at MajorBio Bio‐Pharm Technology Co., Ltd. (Shanghai, China) using the NovaSeq X Plus platform. Raw data were uploaded to the NCBI SRA database (BioProject accession number: PRJNA1321590).

To ensure the accuracy and high quality of sequencing data, the raw reads were filtered and analyzed. Clean reads were obtained by fastp [20]. Clean reads were compared with the reference genome (GCF_020184175.1) of *A. gossypii*. Hisat2 [26] was used for alignment to the reference genome. Mapped reads were assembled into transcripts using StringTie [27]. To identify DEGs (differentially expression genes) between two different samples, the expression level of each transcript was calculated according to the transcripts per million reads (TPM) method. RSEM [28] was used for quantitative analysis of genes or transcripts. Differentially expressed genes were identified using DESeq2 [29] with the following thresholds: |log2FC| ≥ 1 and FDR < 0.05. In addition, functional‐enrichment analysis including GO and KEGG was performed to identify which DEGs were significantly enriched in GO terms and metabolic pathways at Bonferroni‐corrected *p*‐value < 0.05 compared with the whole‐transcriptome background. GO functional enrichment and KEGG pathway analysis were carried out by Goatools [30] and Python scipy software, respectively.

### 2.8 Detection of *D. tsuruhatensis* Copy Number and Salicylic Acid Synthesis Gene Expression

To evaluate the uptake efficiency of *D. tsuruhatensis* by cotton plants and the influence of aphid infestation on bacterial abundance, cotyledon‐stage plants were placed with their roots immersed in centrifuge tubes containing either sterile water or a bacterial suspension. The experiment included four treatment groups: 1) plants treated with sterile water and without aphid infestation; 2) plants treated with sterile water and subjected to aphid infestation; 3) plants treated with *D. tsuruhatensis* suspension and without aphid infestation; and 4) plants treated with *D. tsuruhatensis* suspension and subjected to aphid infestation. Plant samples were collected at 3, 12, 24, and 48 h after treatment for subsequent DNA and RNA extraction.

DNA extracted from these samples was used to quantify *D. tsuruhatensis* copy number as described in the previous section. Total RNA was isolated for assessing the expression levels of SA biosynthesis‐related genes in cotton, following the method outlined earlier. cDNA was synthesized from 1 µg of total RNA per sample using the PrimeScript RT Reagent Kit with gDNA Eraser (Takara, Dalian, China) according to the manufacturer's instructions. Quantitative PCR was performed using the same thermal profile and reaction setup as applied for *D. tsuruhatensis* copy number detection. *GhUBI1* (GenBank accession number: EU604080) was used as an internal reference gene for normalization. Primer sequences are provided in Table S2.

### 2.9 Effects of *D. tsuruhatensis* and RNAi (*UGT2B17*) on the Fitness of Cotton Aphids

To evaluate the fitness of cotton aphids under different treatments, four experimental groups of cotton plants were established: 1) plants treated with sterile water; 2) plants treated with *D. tsuruhatensis* suspension; 3) plants treated with sterile water and infested with aphids subjected to ds*UGT2B17* treatment; and 4) plants treated with *D. tsuruhatensis* suspension and infested with aphids subjected to ds*UGT2B17* treatment.

Cotyledon‐stage live plants were used, with their roots immersed in centrifuge tubes containing either sterile water or bacterial suspension. *D. tsuruhatensis* was cultured in R2A liquid medium to an OD_600_ of 0.8, harvested by centrifugation at 3000 rpm, and resuspended in an equal volume of sterile water. Based on transcriptomic data, the gene *UGT2B17*, implicated in detoxification metabolism of cotton aphids and suppressed by *D. tsuruhatensis*, was selected for functional analysis. Double‐stranded RNA (dsRNA) targeting *UGT2B17* was synthesized using the T7 RNAi Transcription Kit (Vazyme, China) according to the manufacturer's instructions, with primers listed in Table S2. The dsRNA was diluted to 1000 ng/µL using 0.5 mol/L sucrose solution. One‐day‐old adult aphids were subjected to RNAi via feeding with the dsRNA‐sucrose solution for two days and then transferred to cotton leaves. Control aphids were fed 0.5 mol/L sucrose only for the same duration. Each treatment included 60 individual aphids. Mortality and fecundity were recorded daily. After two days of RNAi treatment, aphids were collected to assess interference efficiency using qRT‐PCR. *EF1α* (*elongation factor 1 alpha*) was used as an internal reference gene for normalization; corresponding primers are listed in Table S2.

Detection of EPG (Electrical Penetration Graph). EPG waveform recordings of aphid feeding behavior were conducted in a controlled environment (26°C ± 1°C, 300 ± 30 lx). The treatment methods were the same as those of the four treatment groups described above in this section. One‐day‐old adult aphids were starved for 4 h prior to testing. Experiments lasted 6 h, with 20 replicates per treatment, and each aphid was used only once. Data acquisition and analysis were performed using DC‐EPG Giga‐8 instrumentation with Style+d and Style+a software [31].

### 2.10 The Direct Effect of *D. tsuruhatensis* on Cotton Aphids

To investigate whether *D. tsuruhatensis* directly inhibits cotton aphids, we performed a direct feeding assay by supplementing the artificial diet (0.5 mol/L sucrose solution) with a bacterial suspension of *D. tsuruhatensis* resuspended in sterile water (OD_600_ = 0.8). The control group was fed only the sucrose solution. One‐day‐old adult cotton aphids were used in the experiment and were alternately fed every 24 h with the artificial diet and healthy cotton leaves. Mortality was recorded daily, and the number of offspring produced per 10 aphids was counted over a period of seven days. Three biological replicates were set for both the treatment and control groups, with 20 aphids per replicate.

### 2.11 Determination of Salicylic Acid and Jasmonic Acid Content

To determine whether *D. tsuruhatensis* inoculation induces SA accumulation in cotton, plants treated with a bacterial suspension were analyzed. *D. tsuruhatensis* was cultured in R2A liquid medium until an OD_600_ of 0.8 was reached. The bacterial cells were then pelleted by centrifugation at 3000 rpm, washed, and resuspended in an equal volume of sterile water. At the cotyledon stage, living plants with their roots immersed in centrifuge tubes containing the bacterial suspension were used as the treatment group, while control plants were exposed to sterile water only. Plant samples were collected at 3, 12, 24, and 48 h post‐treatment, with five biological replicates per time point, each consisting of 10 plants. The levels of SA and JA were quantified using a Plant Salicylic Acid (SA) ELISA Kit and a Plant Jasmonic Acid (JA) ELISA Kit (Beijing Lybdbio Biotechnology Co., Ltd), respectively, following the manufacturer's instructions. Absorbance was measured with a Biotek Synergy2 microplate reader (BioTek Instruments, Inc., Winooski, VT, USA).

### 2.12 Effects of Salicylic Acid on *UGT2B17* Gene Expression and Life Parameters in Cotton Aphids

To specifically examine the influence of SA on the expression of the *UGT2B17* gene in cotton aphids, one‐day‐old adult aphids were fed a 20 µg/mL SA solution (Solarbio, Beijing, China, Cat: S7080, AR) prepared in 0.5 mol/L sucrose, using a double‐ended glass tube feeding system. The control group received 0.5 mol/L sucrose solution only. One end of the tube was sealed with Parafilm (Item No: PM996, Bemis) to allow feeding for two days, while the opposite end was covered with perforated plastic film. Each treatment involved processing 60 aphid individuals. After feeding, aphids were flash‐frozen in liquid nitrogen for subsequent RNA extraction, cDNA synthesis, and qRT‐PCR analysis of *UGT2B17* expression, as described in previous sections. *EF1α* [32] was used as an internal control for normalization; primer sequences were listed in Table S2.

Effects of SA on life table parameters and population growth of cotton aphids. The SA solution at a concentration of 20 µg/mL was applied evenly onto cotton leaves each day, with leaves being replaced daily. One‐day‐old adult aphids were used in the experiment. Individual aphids were monitored daily for molting, mortality, and reproduction. Life table parameters, including the net reproductive rate (*R_0_*), intrinsic rate of increase (*r*), finite rate of increase (*λ*), and mean generation time (*T*), were calculated using TWOSEX‐MSChart software [33].

### 2.13 Phylogenetic Analysis and Protein Sequence Domain Prediction

The nucleic acid sequences of *UDP‐glucosyltransferase* and *D. tsuruhatensis* were obtained from the NCBI database, with the accession numbers listed after the species names. A phylogenetic tree was constructed using MEGA7 software with the neighbor‐joining method with 1,000 bootstrap replicates. Conserved domain analysis of the protein sequences was performed using Conserved Domain Search (CD‐Search).

## 3 Results

### 3.1 The Effect of *A. gossypii* on Bacteria in Cotton

**FIGURE 1:**
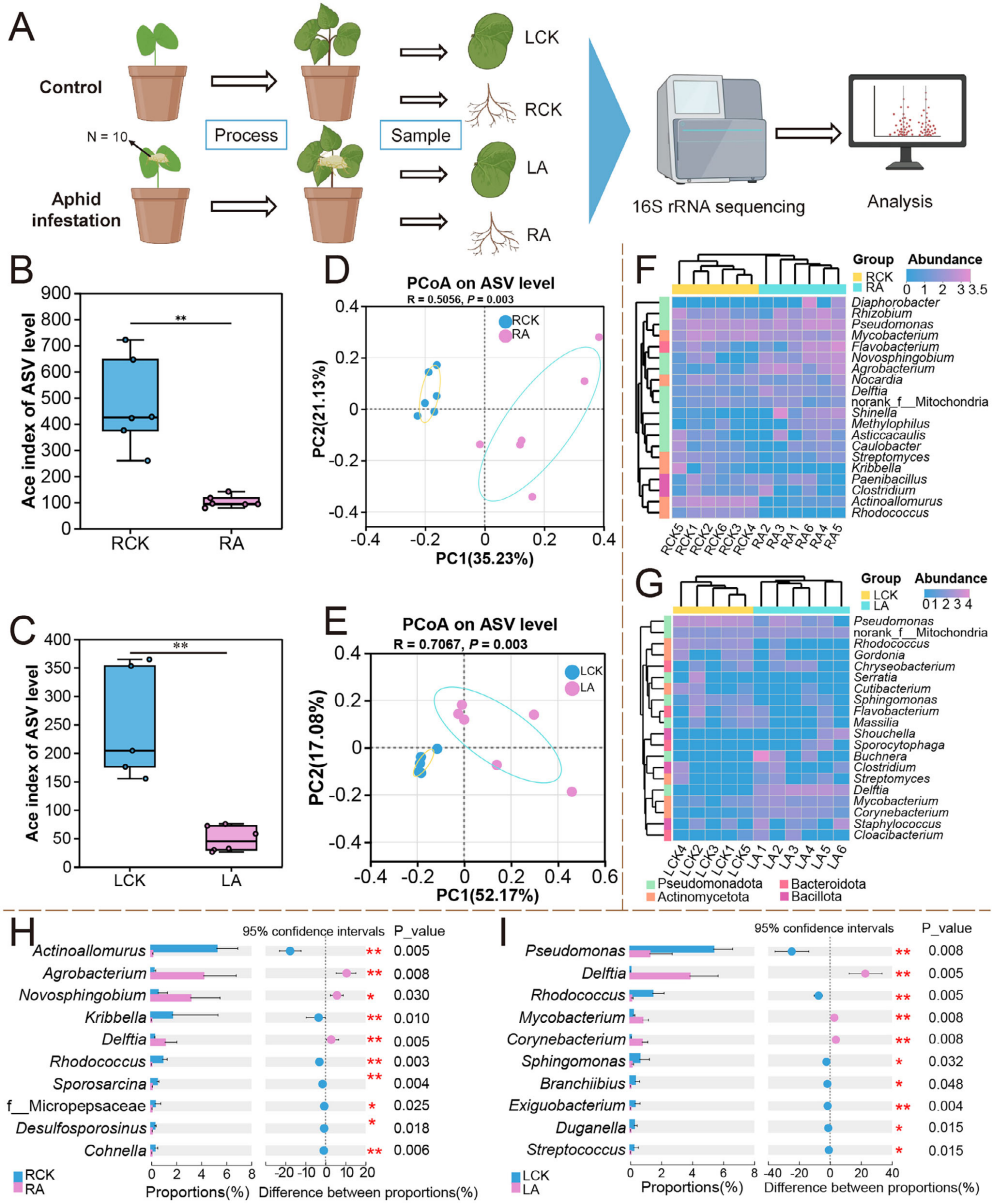
Effects of cotton aphid infestation on the bacterial community in cotton. (A) Experimental process and apparatus. (B,C) ACE index at the ASV level in cotton roots (B) and leaves (C). Differences were assessed using a student's *t*‐test, with ^**^ indicating 0.001 < *p* < 0.01. (D,E) PCoA of bacterial communities at the ASV level in roots (D) and leaves (E), based on the abund_jaccard distance algorithm. Group differences were tested using ANOSIM. *P* < 0.05 indicates a significant difference. (F,G) Cluster heatmaps at the genus level for roots (F) and leaves (G). (H,I) Differential abundance analysis of the top 10 significantly different bacterial genera in roots (H) and leaves (I). Student's *t*‐test was applied, with ^*^ indicating 0.01 < *p* < 0.05 and ^**^ indicating 0.001 < *p* < 0.01. RCK: roots from aphid‐free plants; RA: roots from aphid‐infested plants; LCK: leaves from aphid‐free plants; LA: leaves from aphid‐infested plants.

The test procedure is shown in Figure 1A. Aphid infestation significantly reduced the ACE index of bacterial communities in both cotton roots (Figure 1B) and leaves (Figure 1C). Significant differences in bacterial community composition were observed between infested and control groups in roots (Figure 1D, *P* = 0.003) and leaves (Figure 1E, *P* = 0.003), primarily reflected by separation along the first principal coordinate (PC1). Differential abundance analysis revealed enrichment of four major phyla: Pseudomonadota, Actinomycetota, Bacteroidota, and Bacillota (Figure 1F,G), with Pseudomonadota showing the greatest enrichment. Cluster analysis clearly distinguished between aphid‐infested and uninfested samples in both roots and leaves. Notably, among the top 10 differentially abundant genera, *Delftia* showed significantly increased relative abundance in both roots (Figure 1H, *p* = 0.005) and leaves (Figure 1I, *p* = 0.005) of aphid‐infested plants.

### 3.2 Isolation, Identification, and Insecticidal Activity Assay of Bacteria From Cotton

**FIGURE 2:**
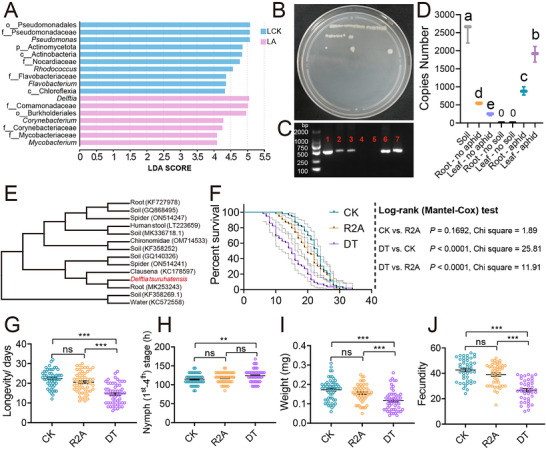
Soil‐derived *D. tsuruhatensis* reduces cotton aphid fitness. (A) LDA analysis of differentially abundant bacterial genera in leaves. (B) Colony morphology of *D. tsuruhatensis*. (C) PCR electrophoresis detection of *D. tsuruhatensis*. Lanes indicate sample sources: 1: soil; 2: root from aphid‐free greenhouse‐grown cotton; 3: leaf from aphid‐free greenhouse‐grown cotton; 4: root from tissue‐cultured cotton; 5: leaf from tissue‐cultured cotton; 6: root from aphid‐infested greenhouse‐grown cotton; 7: leaf from aphid‐infested greenhouse‐grown cotton. (D) Absolute quantification of *D. tsuruhatensis* in samples from (C). Different letters indicate significant differences (*p* < 0.05) based on one‐way ANOVA and Duncan's test. (E) Phylogenetic analysis of *D. tsuruhatensis*. Numbers represent NCBI accession numbers. (F) Survival curves of aphids treated with *D. tsuruhatensis*. CK: sterile water control; R2A: R2A medium control; DT: *D. tsuruhatensis* treatment. Statistical significance was determined using the log‐rank (Mantel–Cox) test (*p* < 0.05). (G–J) Effects of *D. tsuruhatensis* on aphid longevity (G), nymphal duration (H), weight of fourth‐instar nymphs (I), and the number of offspring (J). Significance was determined by student's *t*‐test: ^**^ for 0.001< *p* < 0.01, ^***^ for *p* < 0.0001; ns: not significant.

LDA identified *Delftia* in the leaves as a signature genus with significant influence on community differences (Figure 2A). A total of 41 bacterial isolates were obtained from cotton roots and leaves, among which *D. tsuruhatensis* was identified (Table S1). Colonies of *D. tsuruhatensis* appeared white and smooth on agar plates (Figure 2B). No PCR amplification bands were observed in lanes 4 and 5 (Figure 2C), indicating that the cotton itself did not contain *D. tsuruhatensis*. Notably, stronger bands were detected in both roots and leaves of aphid‐infested plants compared to uninfested controls (Figure 2C), a finding corroborated by bacterial copy number quantification (Figure 2D). Phylogenetic analysis revealed that the cotton‐derived *D. tsuruhatensis* strain was most closely related to isolates from plant roots (Figure 2E).

Insecticidal activity screening of the 41 isolated bacteria showed that *D. tsuruhatensis* significantly reduced aphid fitness. Survival rate in the DT group was significantly lower than in the R2A (*p* < 0.0001) and control (CK, *p* < 0.0001) groups, while no significant difference was observed between the CK and R2A groups (*p* = 0.1692) (Figure 2F). *D. tsuruhatensis* treatment significantly shortened aphid longevity (Figure 2G) and prolonged the nymphal stage (Figure 2H). Additionally, fourth‐instar aphid weight was significantly reduced in the DT group (Figure 2I), and nutritional deficiency led to a significant decline in fecundity (Figure 2J).

### 3.3 *D. tsuruhatensis* Significantly Inhibits the Expression of the *UGT2B17* in Cotton Aphids

**FIGURE 3:**
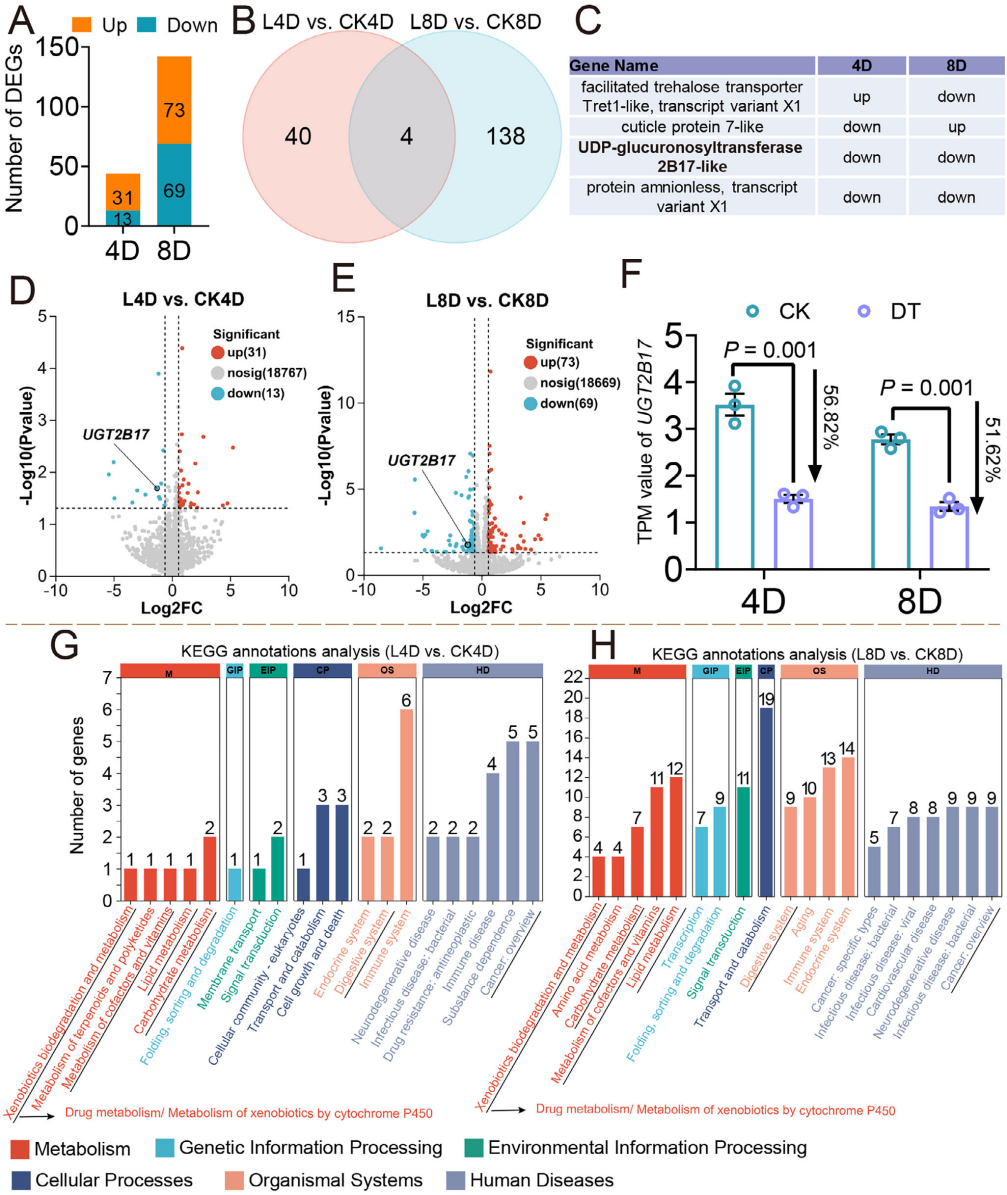
Transcriptomic response of cotton aphids to *D. tsuruhatensis* infection. (A) Summary of differentially expressed genes (DEGs). (B) Venn diagram showing unique and shared DEGs between two comparison groups. (C) Common DEGs across both groups. “Up” indicates significantly upregulated genes; “down” indicates significantly downregulated genes. (D,E) Volcano plots of DEGs. (F) Significantly reduced expression of *UGT2B17*; significance was determined by student's *t*‐test (*p* < 0.05). (G,H) KEGG enrichment analysis of DEGs. The black underline highlights the pathway to which *UGT2B17* was annotated. 4D (L4D): aphids treated with *D. tsuruhatensis* for 4 days; 8D (L8D): aphids treated with *D. tsuruhatensis* for 8 days; CK4D: control aphids treated with sterile water for 4 days; CK8D: control aphids treated with sterile water for 8 days; DT: treatment with *D. tsuruhatensis*.

Based on the bioassay results, transcriptome analysis of the cotton aphid was performed at two critical time points: day 8 (point of sharp decline in aphid survival) and day 4 (mid‐point). *D. tsuruhatensis* treatment resulted in 44 and 142 DEGs on days 4 and 8, respectively (Figure 3A), with four DEGs shared between the two time points (Figure 3B). Among these, the gene encoding *UGT2B17*, which is associated with insect detoxification metabolism, was significantly suppressed (Figure 3C–E). Expression of *UGT2B17* was downregulated by 56.82% and 51.62% at 4 and 8 days, respectively, following DT treatment (Figure 3F). KEGG functional annotation revealed that *UGT2B17* was significantly enriched in the “Xenobiotics biodegradation and metabolism” pathway (indicated by black underlining), which is closely associated with drug metabolism (Figure 3G,H).

### 3.4 Combined Insecticidal Efficacy of *D. tsuruhatensis* and RNAi (*UGT2B17*)

Phylogenetic analysis indicated that the *UGT2B17* gene in the cotton aphid is most closely related to its ortholog in *Diuraphis noxia* (Figure S1A). Domain prediction further revealed that *UGT2B17* contains functional domains characteristic of UDP‐glucosyltransferases, consistent with KEGG annotation results, underscoring its role in resistance to xenobiotics and importance in detoxification metabolism and immune function (Figure S1B).

**FIGURE 4:**
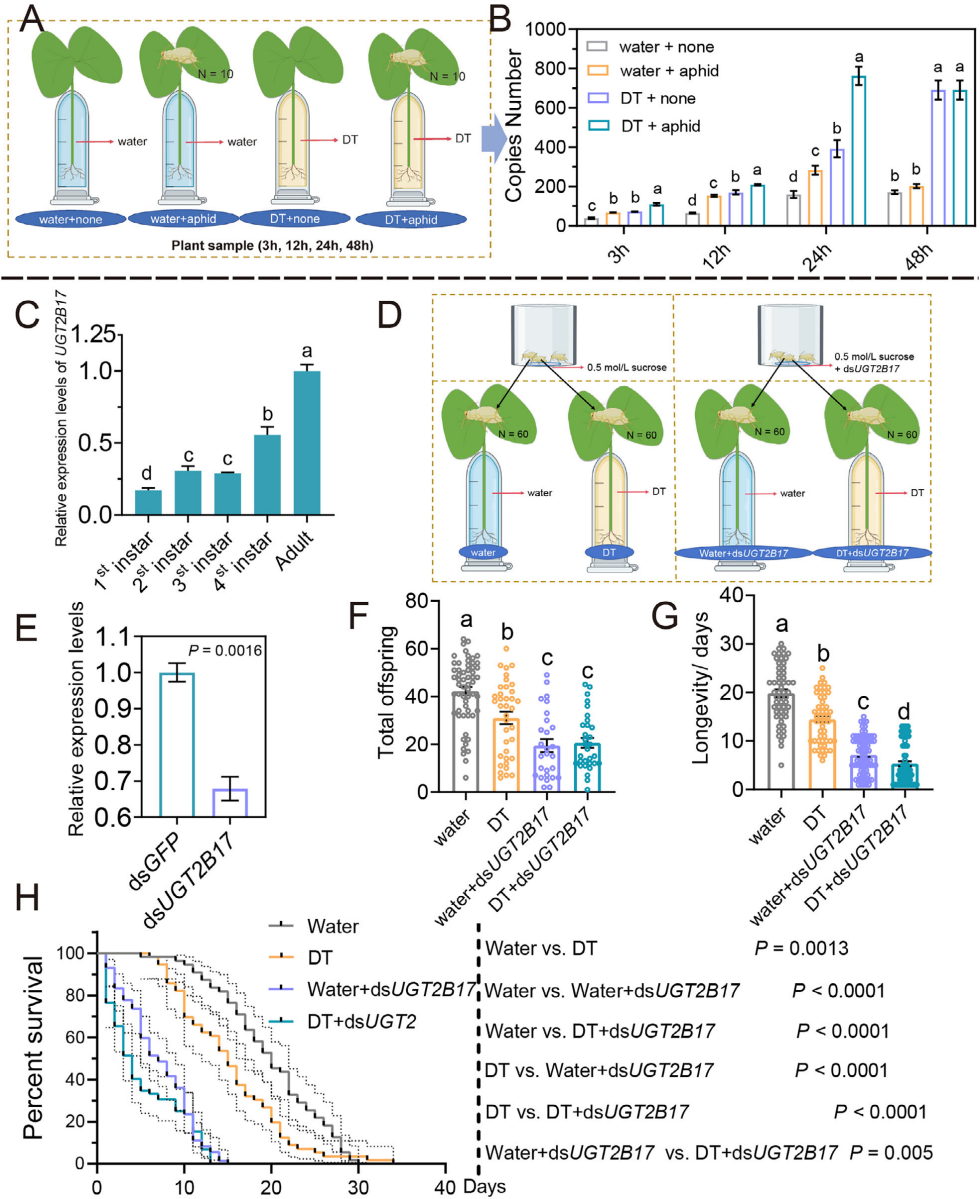
Combined RNAi and *D. tsuruhatensis* treatment reduces cotton aphid fitness. (A) From left to right, the treatments were water, water with aphids, DT, and DT with aphids, with 10 aphids per treatment. Cotton samples were collected at 3, 12, 24, and 48 h after treatment. (B) Quantification of *D. tsuruhatensis* copy number. (C) Expression levels of *UGT2B17* across different developmental stages of the cotton aphid. (D) Experimental design for bioassays. (E) Efficiency of RNAi‐mediated knockdown of *UGT2B17*. (F,G) Total number of offspring and longevity of aphids under various treatments. (H) Survival rates of aphids; significance was determined by the log‐rank (Mantel–Cox) test (*p* < 0.05). Different letters indicate significant differences (*p* < 0.05) based on one‐way ANOVA followed by Duncan's test. Pairwise comparisons were performed using student's *t*‐test (*p* < 0.05). Treatment groups: water+none: plants supplied with sterile water, no aphid infestation; water+aphid: plants supplied with sterile water, with aphid infestation; DT+none: plants supplied with *D. tsuruhatensis* suspension, no aphid infestation; DT+aphid: plants supplied with *D. tsuruhatensis* suspension, with aphid infestation.

Bacterial counts measured by qPCR indicated that cotton plants can enhance the abundance of *D. tsuruhatensis* on leaves when their roots are placed in medium containing *D. tsuruhatensis* (Figure 4A,B). Aphid infestation further enhanced bacterial abundance (Figure 4B). The combined stimulation from both bacterial application and aphid feeding resulted in the most rapid accumulation of *D. tsuruhatensis* in cotton leaves (Figure 4B). The expression of *UGT2B17* increased throughout aphid development, reaching the highest level in adults (Figure 4C). To evaluate the combined insecticidal effect of *D. tsuruhatensis* and RNAi, bioassays were conducted using the validated experimental system established in Figure 4D. dsRNA‐mediated knockdown significantly reduced *UGT2B17* expression (*p* = 0.0016, Figure 4E). Notably, both DT and ds*UGT2B17* treatments significantly reduced the total number of offspring (Figure 4F) and longevity of aphids (Figure 4G), with a pronounced synergistic effect on reducing overall fitness. Similarly, compared to the water control, DT and ds*UGT2B17* treatments significantly decreased aphid survival. Strong insecticidal effects were observed as early as the second day in the water+ds*UGT2B17* and DT+ds*UGT2B17* groups (Figure 4H).

**FIGURE 5:**
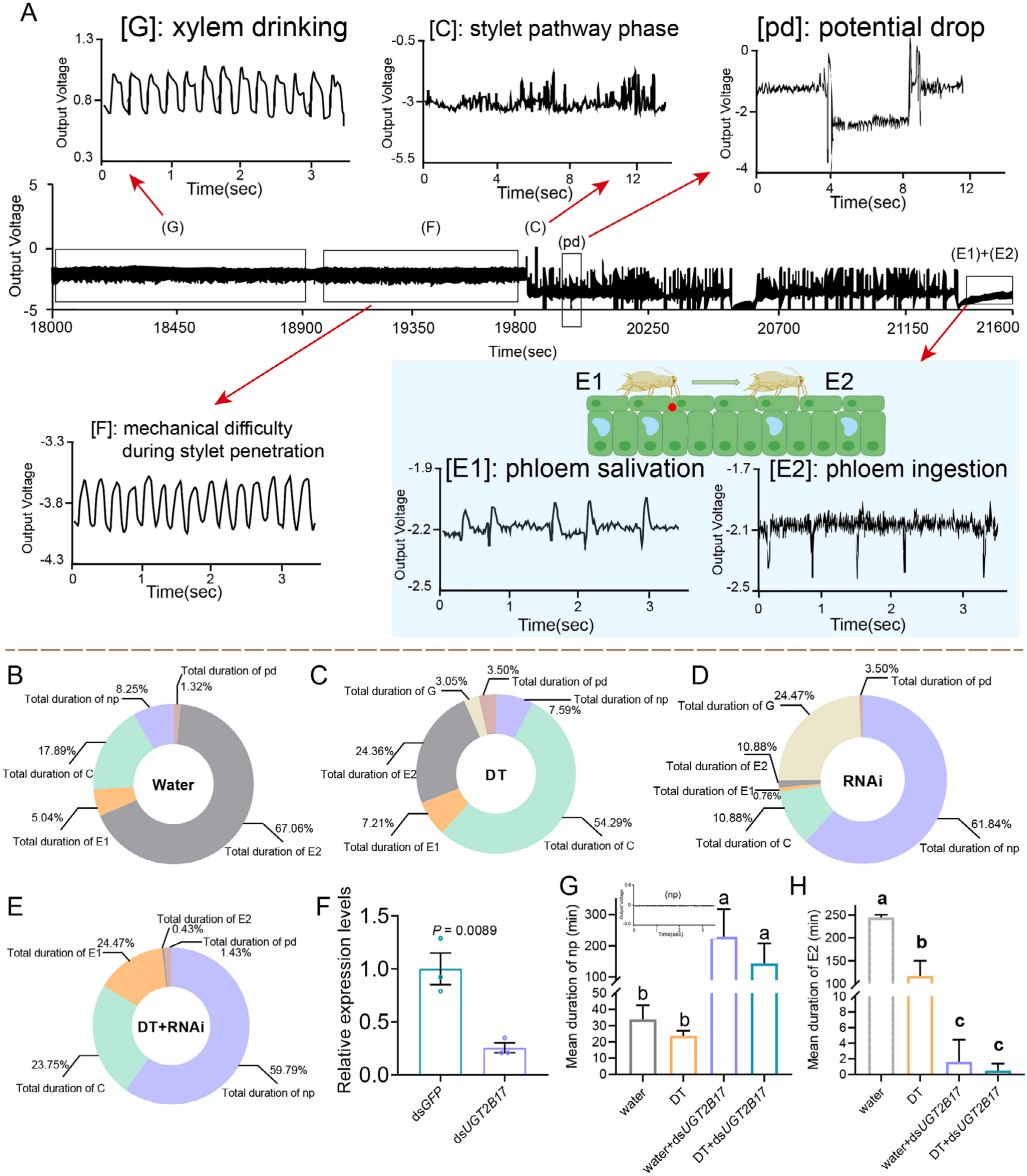
EPG analysis of feeding behaviors of cotton aphids. (A) Typical EPG waveform of aphids feeding on cotton leaves. The waveforms were classified as follows: [G]—xylem drinking; [C]—stylet pathway phase; [pd]—potential drop (analyzed separately within the [C] phase); [F]—mechanical difficulty during stylet penetration; [E1]—phloem salivation; [E2]—phloem ingestion; [np]—non‐penetration (The waveform is a straight line). (B) Analysis of the proportion of different feeding behaviors of cotton aphids on sterile water. (C) Analysis of the proportion of different feeding behaviors of cotton aphids on DT. (D) Analysis of the proportion of different feeding behaviors of cotton aphids on RNAi. (E) Analysis of the proportion of different feeding behaviors of cotton aphids on DT + RNAi. (F) Relative expression of the *UGT2B17* gene after RNAi. Student's *t*‐test (*p* < 0.05). (G) Comparison of the mean time of np waves of cotton aphids across four treatments (One‐way ANOVA). (H) Comparison of the mean time of E2 waves of cotton aphids across four treatments (Kruskal‐Wallis test). Different letters indicate significant differences (*p* < 0.05).

EPG recordings were conducted to monitor the feeding behavior of cotton aphids. Representative EPG waveforms and its enlarged view of aphid feeding on cotton leaves were shown in Figure 5A. In the sterile water control group, aphids exhibited the longest duration of E2 waveform, indicating sustained phloem feeding (Figure 5B). Under DT treatment, aphids showed prolonged C waveform activity, reflecting continuous probing with reduced feeding time (Figure 5C). In the RNAi group, aphids largely ceased feeding and probing, resulting in the longest np phase (Figure 5D). Similarly, the RNAi+water group displayed extended non‐feeding behavior (Figure 5E). Knockdown of *UGT2B17* via RNAi significantly reduced its expression (Figure 5F). The duration of the np phase was significantly increased in both water+ds*UGT2B17* and DT+ds*UGT2B17* groups (Figure 5G). Furthermore, E2 phase duration was significantly shortened in aphids treated with either DT or ds*UGT2B17* (Figure 5H).

### 3.5 *D. tsuruhatensis* Activates the Salicylic Acid Synthesis Pathway in Cotton

**FIGURE 6:**
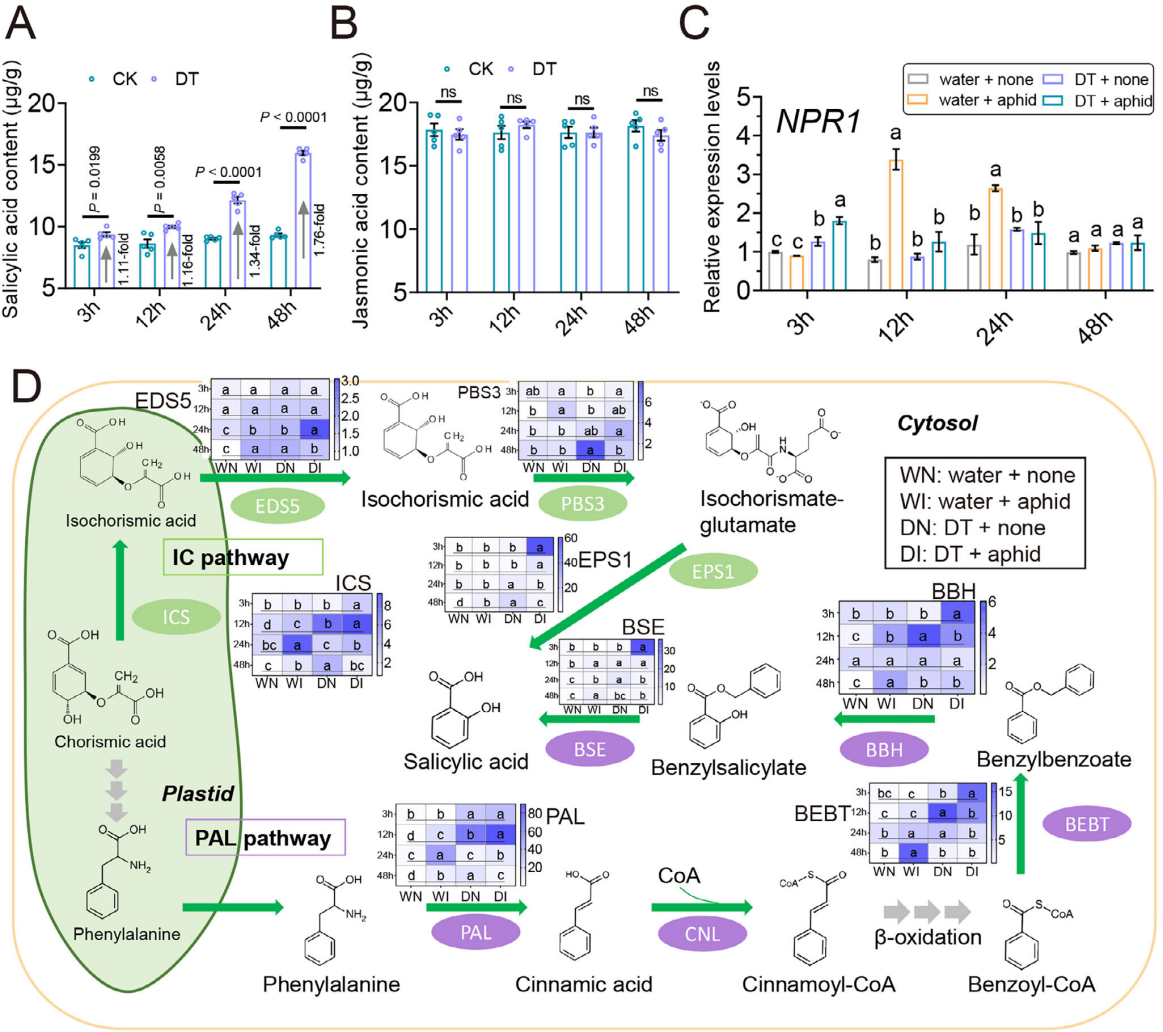
*D. tsuruhatensis* activates salicylic acid biosynthesis in cotton. (A,B) Content levels of SA (A) and JA (B). CK: sterile water control; DT: *D. tsuruhatensis* treatment. Significance was determined by student's *t*‐test (^*^, 0.01< *p* < 0.05, ^**^, 0.001< *p* < 0.01; ns: not significant). (C,D) Expression analysis of SA biosynthesis‐related genes. Different letters indicate significant differences (*p* < 0.05) based on one‐way ANOVA followed by Duncan's test. PAL: Phenylalanine ammonia‐lyase; CNL: CoA ligases; BEBT: Benzyl alcohol benzoyltransferase; BBH: Benzylbenzoate hydroxylase; BSE: Benzylsalicylate esterase; ICS: Isochorismate synthase; EDS5: Enhanced disease susceptibility 5; PBS3: PphB susceptible 3 (4‐substituted benzoates‐glutamate ligase GH3.12). EPS1: Enhanced pseudomonas susceptibility 1.

Piercing‐sucking insects are known to activate SA‐dependent defense pathways in plants. Further investigation demonstrated that treatment with *D. tsuruhatensis* alone significantly increased SA levels in cotton leaves at 3, 12, 24, and 48 h post‐treatment (Figure 6A), whereas no significant change was observed in JA content (Figure 6B), with both phytohormones quantified by ELISA. The non‐expressor of pathogenesis‐related genes 1 (*NPR1*) is a key regulator in insect‐induced SA biosynthesis. At 3 h, both the DT and DT+aphid groups showed significantly elevated *NPR1* expression. By 12 and 24 h, aphid infestation alone resulted in the highest upregulation of *NPR1*, while the DT+aphid group still exhibited an upward trend compared to the control (Figure 6C). Detection of the gene expression levels of two major SA biosynthesis pathways—the isochorismate (IC) and phenylalanine ammonia‐lyase (PAL) pathways—revealed that both *D. tsuruhatensis* treatment and aphid feeding significantly up‐regulated, or induced an increasing trend in, the expression of pathway‐related genes at various time points between 3 and 48 h (Figure 6D).

### 3.6 Salicylic Acid Suppressed Cotton Aphid *UGT2B17* Expression, Thereby Reducing Its Fitness

**FIGURE 7:**
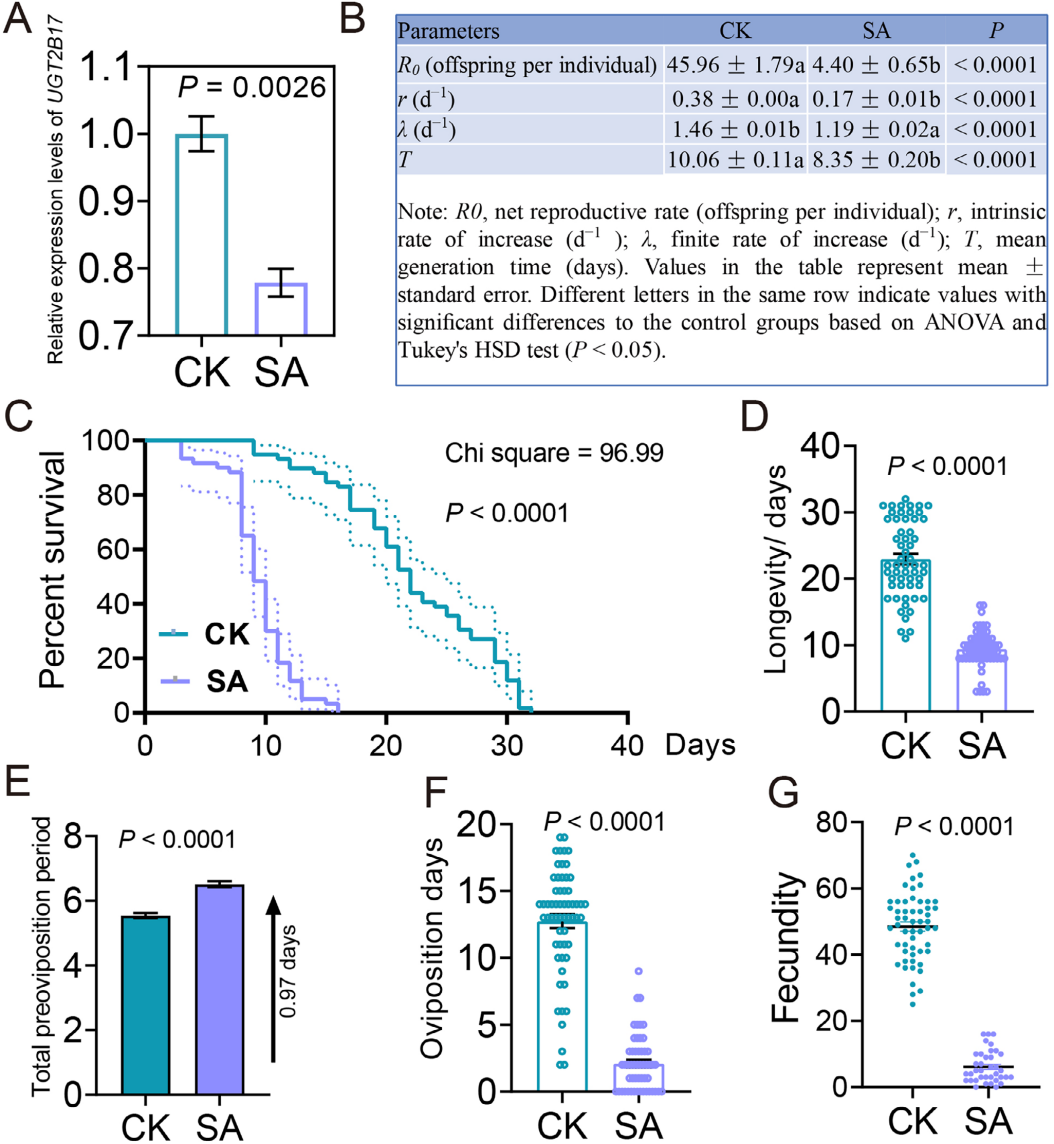
Salicylic acid suppresses cotton aphid population growth. (A) SA significantly downregulates the expression of *UGT2B17*. (B) SA markedly reduces key life‐table parameters of the cotton aphid. (C–G) Effects of SA treatment on aphid survival rate (C), longevity (D), pre‐reproductive period (E), total reproduction time (F), and the number of offspring (G). Survival curves were compared using the log‐rank (Mantel–Cox) test (*p* < 0.05). Pairwise comparisons were performed using student's *t*‐test (*p* < 0.05).

We excluded the possibility that *D. tsuruhatensis* directly inhibits cotton aphids by supplementing the artificial diet with this bacterium. Direct feeding of *D. tsuruhatensis* had no significant effect on *UGT2B17* expression (Figure S2A), survival rate (Figure S2B), or fecundity (Figure S2C) of cotton aphids. Feeding on SA significantly suppressed the expression of the *UGT2B17* gene in cotton aphids, indicating that cotton modulates aphid detoxification gene expression through SA‐mediated defense (Figure 7A). SA treatment led to significant reductions in the *R_0_*, *r*, *λ*, and *T*, indicating strong inhibition of population growth (Figure 7B). Additionally, SA significantly reduced the survival rate compared to the control (*p* < 0.0001; Figure 7C) and shortened aphid longevity (Figure 7D). Furthermore, aphids exposed to SA exhibited a prolonged pre‐reproductive period by 0.97 days (Figure 7E), together with significant decreases in the duration of oviposition (Figure 7F) and overall fecundity (Figure 7G).

## 4 Discussion

Although plants have evolved intrinsic adaptive mechanisms to mitigate most biotic and abiotic stresses in nature, they also rely on their microbial partners for survival and defense against invaders [34]. Since ancestral plants colonized land 450 million years ago, interactions between plants and their associated microbes have formed a collective meta‐organism often referred to as the “holobiont” [6]. The soil is a primary reservoir of plant‐associated microbes, yet the mechanistic basis of how soil bacteria participate in plant defense against aphids remains unclear. This study elucidates a significant ecological phenomenon: cotton plants recruit the beneficial symbiotic bacterium *D. tsuruhatensis* from the soil in response to aphid infestation. Further experiments reveal a novel molecular mechanism whereby the bacterium activates SA‐dependent defense signaling in the plant, leading to suppression of the aphid detoxification gene *UGT2B17*, ultimately reducing aphid fitness. We also demonstrate a synergistic effect between *D. tsuruhatensis* and RNA interference (RNAi) targeting *UGT2B17*, providing a robust theoretical and experimental foundation for developing innovative green strategies for aphid control based on combined microbial and RNAi treatments.

The assembly of plant‐associated bacterial communities is not stochastic but governed by specific ecological rules [35, 36]. The plant immune system, including defense hormones such as SA, serves as a major driver in shaping the root microbiome by selectively recruiting microbes from the available soil pool [37]. Herbivory by insects triggers plant defense hormones, and aphid feeding, in particular, can promote rhizobacteria (e.g., *Bacillus* and *Pseudomonas* spp.) that induce systemic resistance (ISR), activate immune responses, and enhance plant health [38, 39]. Our study identified and validated the enrichment of the beneficial bacterium *D. tsuruhatensis* in both roots and leaves of cotton plants following aphid attack, supporting an ecological model in which plants recruit soil bacteria for defense.

Endophytic microbes can enhance host plant tolerance to stress by stimulating the synthesis of secondary metabolites. Plants recognize specific molecules released by microbial communities, which trigger signaling networks, modulate gene expression, and promote accumulation of defensive compounds [40]. Salicylic acid is a key immune signal in plant resistance to pests. While its biosynthesis via the IC and PAL pathways is well established [41, 42], our study demonstrates that elevated abundance of *D. tsuruhatensis* upregulates genes in both SA biosynthesis pathways, leading to significantly increased SA levels. However, sustained SA accumulation can adversely affect plant fitness [43], which may explain the moderate SA induction and variable expression patterns of SA‐related genes observed in our study, reflecting dynamic regulation within the pathway. Furthermore, both aphid feeding and *D. tsuruhatensis* inoculation induced high expression of *NPR1*, a central regulator of SA signaling. *NPR1* perception of SA is essential for activating defense‐related genes and establishing systemic acquired resistance [44, 45].

On the other hand, aphid stylet penetration activates SA‐dependent defenses [46]. Increased SA levels in the phloem of infested plants can inhibit aphid feeding [47], potentially by facilitating tissue repair or disrupting nutrient assimilation [48]. Our EPG and life‐table analyses revealed that cotton plants treated with *D. tsuruhatensis* significantly reduced aphid phloem ingestion, leading to prolonged nymphal development, reduced fecundity, and lower survival—phenotypes mirrored in SA‐treated aphids, confirming that SA‐mediated signaling is central to the bacterium‐induced defense.

The evolutionary arms race between plants and herbivores has driven the expansion of detoxification enzyme families such as UDP‐glycosyltransferases (UGTs) in insects [49, 50]. UGTs are major phase II enzymes involved in the detoxification of endo‐ and xenobiotics. Although most aphid UGT studies have focused on insecticide resistance [51, 52], our work highlights the critical role of *UGT2B17* in cotton aphid counter‐defense against plant SA. Silencing *UGT2B17* significantly reduced aphid survival and reproduction. Our results showed that the expression level of *UGT2B17* was significantly reduced in cotton aphids fed with SA, and prolonged or high‐level exposure to SA may cause direct physiological damage to the aphids. Although most current studies focus on SA‐mediated plant defense against pests by inducing insecticidal secondary metabolites, altering nutrient composition, or attracting natural enemies, several studies have demonstrated the direct defensive effect of SA, leading to reduced aphid fitness [53]. However, the mechanism by which SA suppresses *UGT2B17* expression remains unclear. We hypothesize that: 1) SA or its derivatives may directly inhibit aphid detoxification mechanisms; or 2) SA‐induced feeding suppression may lead to nutritional deficiency and compromised detoxification capacity. These hypotheses represent promising directions for future research.

This is the first study to report the protective role of *D. tsuruhatensis* in cotton against aphids. Previous research on its insecticidal activity remains limited. Our study elucidates a sophisticated defense strategy wherein cotton plants, under aphid attack, actively recruit the soil‐borne beneficial bacterium *D. tsuruhatensis*. This bacterium primes systemic SA‐dependent defenses in the host, which remarkably extend to suppressing a key detoxification gene (*UGT2B17*) within the aphid pest itself, exemplifying a novel form of cross‐kingdom gene regulation in plant‐herbivore interactions.

## 5 Conclusion

**FIGURE 8:**
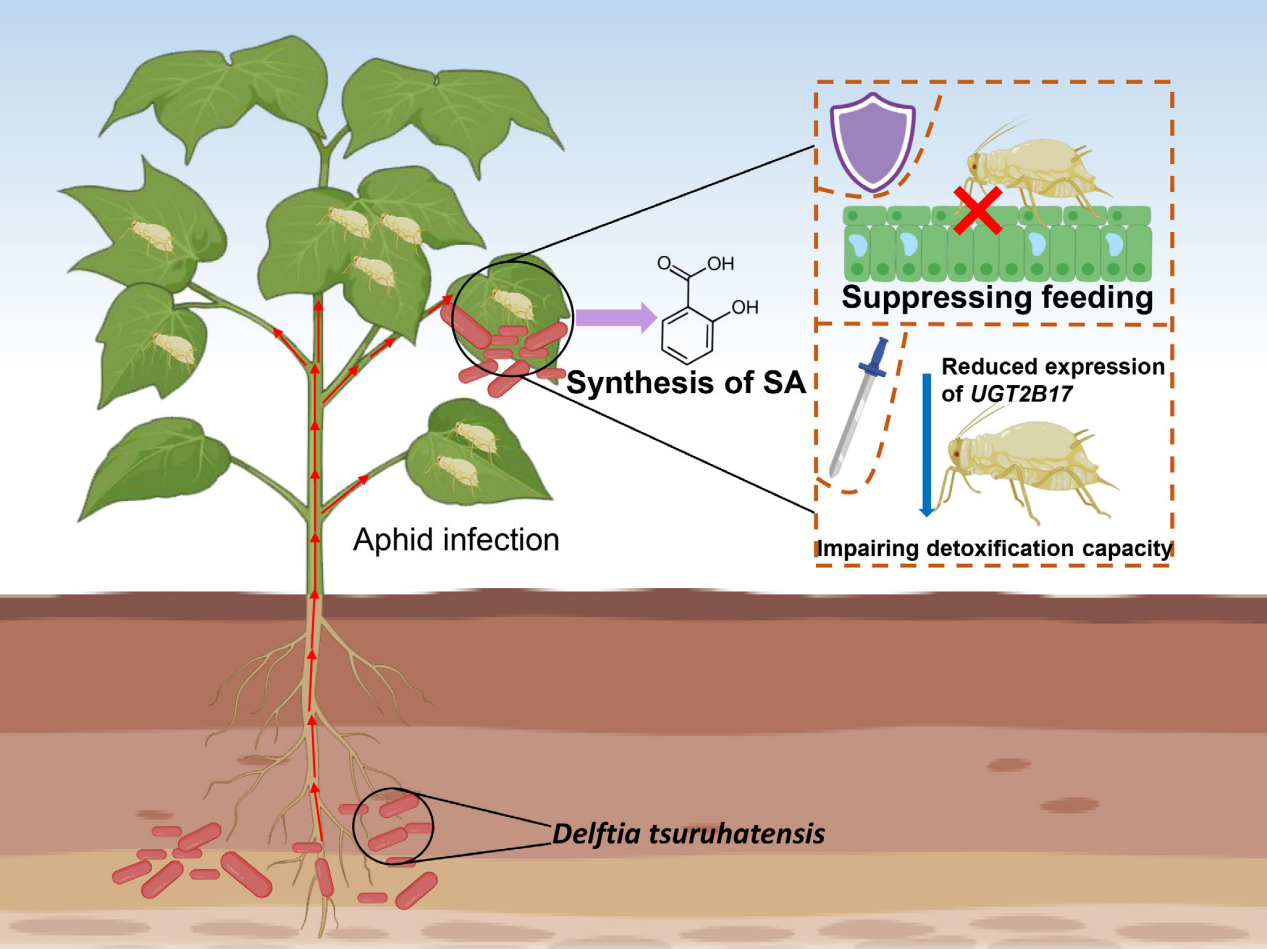
Cotton recruits the beneficial soil bacterium *D. tsuruhatensis* to defend against cotton aphid infestation.

This study systematically reveals a complex interactive network involving plants, microbes, and insects, elucidating the ecological and molecular mechanisms by which cotton enhances its resistance to aphids through the active recruitment of the beneficial soil bacterium *D. tsuruhatensis* (Figure 8). Our work advances the holobiont concept by revealing how plants leverage soil microbiomes for targeted pest suppression, while the combinatorial effect of *D. tsuruhatensis* and RNAi (*UGT2B17*) presents a translatable blueprint for sustainable agriculture that bridges microbial ecology and RNAi‐based pest control. Moreover, this study provides a robust theoretical foundation and practical avenues for developing novel strategies for the sustainable management of agricultural pests.

## Author Contributions

H.X. preparation of the manuscript. H.X. and F.C.D. performed the laboratory experiments and analysed the data. Q.Q.Y., X.Z.Z., T.F.Z., L.W., and K.X.Z. coordinated the study and validated the experimental results. D.Y.L. and J.C.J. coordinated the sample collection. X.K.G., J.Y.L., and J.J.C. contributed to the conceptual design of the study and securing funding support. All authors have read and agreed to the published version of the manuscript.

## Conflicts of Interest

The authors declare no conflicts of interest.

## Supporting information




**Supporting File**: advs75321‐sup‐0001‐SuppMat.docx.

## Data Availability

The data that support the findings of this study are available from the corresponding author upon reasonable request.

## References

[1] P. A. Divekar , S. Narayana , B. A. Divekar , et al., “Plant Secondary Metabolites as Defense Tools Against Herbivores for Sustainable Crop Protection,” International Journal of Molecular Sciences 23, no. 5 (2022): 2690, 10.3390/ijms23052690. 35269836 PMC8910576

[2] S. Mostafa , Y. Wang , W. Zeng , and B. Jin , “Plant Responses to Herbivory, Wounding, and Infection,” International Journal of Molecular Sciences 23, no. 13 (2022): 7031, 10.3390/ijms23137031. 35806046 PMC9266417

[3] Y. Shibata , K. Kawakita , and D. Takemoto , “Age‐Related Resistance of *Nicotiana benthamiana* Against Hemibiotrophic Pathogen *Phytophthora infestans* Requires Both Ethylene‐ and Salicylic Acid–Mediated Signaling Pathways,” Molecular Plant‐Microbe Interactions® 23, no. 9 (2010): 1130–1142, 10.1094/mpmi-23-9-1130. 20687803

[4] M. L. Berens , H. M. Berry , A. Mine , C. T. Argueso , and K. Tsuda , “Evolution of Hormone Signaling Networks in Plant Defense,” Annual Review of Phytopathology 55 (2017): 401–425, 10.1146/annurev-phyto-080516-035544. 28645231

[5] H. Li , S. La , X. Zhang , L. Gao , and Y. Tian , “Salt‐induced Recruitment of Specific Root‐Associated Bacterial Consortium Capable of Enhancing Plant Adaptability to Salt Stress,” The ISME Journal 15, no. 10 (2021): 2865, 10.1038/s41396-021-00974-2. 33875820 PMC8443564

[6] P. Trivedi , J. E. Leach , S. G. Tringe , T. Sa , and B. K. Singh , “Plant–Microbiome interactions: From community assembly to plant health,” Nature Reviews Microbiology 18, no. 11 (2020): 607–621, 10.1038/s41579-020-0412-1. 32788714

[7] H. Wang , F. Zhang , Y. Zhang , M. Wang , Y. Zhang , and J. Zhang , “Enrichment of novel entomopathogenic *Pseudomonas* species enhances willow resistance to leaf beetles,” Microbiome 12 (2024): 169, 10.1186/s40168-024-01884-z. 39252132 PMC11382411

[8] J. F. White , K. L. Kingsley , Q. Zhang , et al., “Review: Endophytic microbes and their potential applications in crop management,” Pest Management Science 75, no. 10 (2019): 2558–2565, 10.1002/ps.5527. 31228333 PMC6771842

[9] M. J. Pozo , J. A. López‐Ráez , C. Azcón‐Aguilar , and J. M. García‐Garrido , “Phytohormones as integrators of environmental signals in the regulation of mycorrhizal symbioses,” New Phytologist 205, no. 4 (2015): 1431–1436, 10.1111/nph.13252. 25580981

[10] C. Gutjahr , “Phytohormone Signaling in Arbuscular Mycorhiza Development,” Current Opinion in Plant Biology 20 (2014): 26–34, 10.1016/j.pbi.2014.04.003. 24853646

[11] C. M. Pieterse , C. Zamioudis , R. L. Berendsen , D. M. Weller , S. C. Van Wees , and P. A. Bakker , “Induced Systemic Resistance by Beneficial Microbes,” Annual Review of Phytopathology 52 (2014): 347–375, 10.1146/annurev-phyto-082712-102340. 24906124

[12] D. A. Bastias , M. A. Martínez‐Ghersa , C. L. Ballaré , and P. E. Gundel , “Epichloë Fungal Endophytes and Plant Defenses: Not Just Alkaloids,” Trends in Plant Science 22, no. 11 (2017): 939–948, 10.1016/j.tplants.2017.08.005. 28923242

[13] D. F. Mou , P. Kundu , L. Pingault , H. Puri , S. Shinde , and J. Louis , “Monocot crop–aphid interactions: Plant resilience and aphid adaptation,” Current Opinion in Insect Science 57 (2023): 101038, 10.1016/j.cois.2023.101038. 37105496

[14] J. Dampc , M. Kula‐Maximenko , M. Molon , and R. Durak , “Enzymatic Defense Response of Apple Aphid *Aphis pomi* to Increased Temperature,” Insects 11, no. 7 (2020): 436, 10.3390/insects11070436. 32664609 PMC7411948

[15] P. Jasrotia , S. Sharma , M. Nagpal , et al., “Comparative transcriptome analysis of wheat in response to corn leaf aphid, *Rhopalosiphum maidis* F. infestation,” Frontiers in Plant Science 13 (2022): 989365, 10.3389/fpls.2022.989365. 36507434 PMC9730506

[16] K. R. Gadhave , S. Gautam , D. A. Rasmussen , and R. Srinivasan , “Aphid Transmission of Potyvirus: The Largest Plant‐Infecting RNA Virus Genus,” Viruses 12, no. 7 (2020): 773, 10.3390/v12070773. 32708998 PMC7411817

[17] S. C. Wooley , D. S. Smith , E. V. Lonsdorf , et al., “Local Adaptation and Rapid Evolution of Aphids in Response to Genetic Interactions with Their Cottonwood Hosts,” Ecology and Evolution 10, no. 19 (2020): 10532–10542, 10.1002/ece3.6709. 33072278 PMC7548174

[18] V. Nalam , J. Louis , and J. Shah , “Plant defense against aphids, the pest extraordinaire,” Plant Science 279 (2019): 96–107, 10.1016/j.plantsci.2018.04.027. 30709498

[19] K. G. Gebretsadik , Z. Liu , J. Yang , et al., “Plant‐Aphid Interactions: Recent Trends in Plant Resistance to Aphids,” Stress Biology 5, no. 1 (2025): 28, 10.1007/s44154-025-00214-z. 40299207 PMC12041410

[20] S. Chen , Y. Zhou , Y. Chen , and J. Gu , “fastp: an Ultra‐fast All‐in‐One FASTQ Preprocessor,” Bioinformatics 34, no. 17 (2018): i884, 10.1093/bioinformatics/bty560. 30423086 PMC6129281

[21] T. Magoč and S. L. Salzberg , “FLASH: Fast Length Adjustment of Short Reads to Improve Genome Assemblies,” Bioinformatics 27, no. 21 (2011): 2957, 10.1093/bioinformatics/btr507. 21903629 PMC3198573

[22] B. J. Callahan , P. J. McMurdie , M. J. Rosen , A. W. Han , A. J. Johnson , and S. P. Holmes , “DADA2: High‐resolution sample inference from Illumina amplicon data,” Nature Methods 13, no. 7 (2016): 581–583, 10.1038/nmeth.3869. 27214047 PMC4927377

[23] E. Bolyen , J. R. Rideout , M. R. Dillon , et al., “Reproducible, Interactive, Scalable and Extensible Microbiome Data Science Using QIIME 2,” Nature Biotechnology 37, no. 8 (2019): 852, 10.1038/s41587-019-0209-9. PMC7015180 31341288

[24] C. Han , C. Shi , L. Liu , et al., “Majorbio Cloud 2024: Update Single‐cell and Multiomics Workflows,” Imeta 3, no. 4 (2024): 217, 10.1002/imt2.217. PMC11316920 39135689

[25] P. D. Schloss , S. L. Westcott , T. Ryabin , et al., “Introducing mothur: Open‐Source, Platform‐Independent, Community‐Supported Software for Describing and Comparing Microbial Communities,” Applied and Environmental Microbiology 75, no. 23 (2009): 7537–7541, 10.1128/aem.01541-09. 19801464 PMC2786419

[26] D. Kim , B. Langmead , and S. L. Salzberg , “HISAT: A fast spliced aligner with low memory requirements,” Nature Methods 12, no. 4 (2015): 357–360, 10.1038/nmeth.3317. 25751142 PMC4655817

[27] M. Pertea , G. M. Pertea , C. M. Antonescu , T. C. Chang , J. T. Mendell , and S. L. Salzberg , “StringTie enables improved reconstruction of a transcriptome From RNA‐seq reads,” Nature Biotechnology 33, no. 3 (2015): 290–295, 10.1038/nbt.3122. PMC4643835 25690850

[28] B. Li and C. N. Dewey , “RSEM: Accurate Transcript Quantification from RNA‐Seq Data With or Without a Reference Genome,” BMC Bioinformatics 12 (2011): 323, 10.1186/1471-2105-12-323. 21816040 PMC3163565

[29] L. Wang , Z. Feng , X. Wang , X. Wang , and X. Zhang , “DEGseq: An R package for identifying differentially expressed genes From RNA‐seq data,” Bioinformatics 26, no. 1 (2010): 136–138, 10.1093/bioinformatics/btp612. 19855105

[30] D. V. Klopfenstein , L. Zhang , B. S. Pedersen , et al., “GOATOOLS: A Python library for Gene Ontology analyses,” Scientific Reports 8 (2018): 10872, 10.1038/s41598-018-28948-z. 30022098 PMC6052049

[31] V. Nalam , J. Louis , M. Patel , and J. Shah , “Arabidopsis‐Green Peach Aphid Interaction: Rearing the Insect, No‐choice and Fecundity Assays, and Electrical Penetration Graph Technique to Study Insect Feeding Behavior,” Bio‐Protocol 8 (2018): 2950, 10.21769/BioProtoc.2950. PMC8328680 34395762

[32] K. Ma , F. Li , Q. Tang , et al., “ *CYP4CJ1*‐mediated gossypol and tannic acid tolerance in *Aphis gossypii* Glover,” Chemosphere 219 (2019): 961–970, 10.1016/j.chemosphere.2018.12.025. 30572243

[33] H. Chi , A. Güncan , A. Kavousi , et al., “TWOSEX‐MSChart: The Key Tool for Life Table Research and Education,” Entomologia Generalis 42 (2022): 845–849, 10.1127/entomologia/2022/1851.

[34] T. R. Turner , E. K. James , and P. S. Poole , “The plant microbiome,” Genome Biology 14, no. 6 (2013): 209, 10.1186/gb-2013-14-6-209. 23805896 PMC3706808

[35] D. Bulgarelli , K. Schlaeppi , S. Spaepen , E. Ver Loren van Themaat , and P. Schulze‐Lefert , “Structure and Functions of the Bacterial Microbiota of Plants,” Annual Review of Plant Biology 64 (2013): 807–838, 10.1146/annurev-arplant-050312-120106. 23373698

[36] B. Reinhold‐Hurek , W. Bünger , C. S. Burbano , M. Sabale , and T. Hurek , “Roots Shaping Their Microbiome: Global Hotspots for Microbial Activity,” Annual Review of Phytopathology 53 (2015): 403–424, 10.1146/annurev-phyto-082712-102342. 26243728

[37] S. L. Lebeis , S. H. Paredes , D. S. Lundberg , et al., “Salicylic acid modulates colonization of the root microbiome by specific bacterial taxa,” Science 349, no. 6250 (2015): 860–864, 10.1126/science.aaa8764. 26184915

[38] F. Francis , C. Then , A. Francis , Y. A. C. Gbangbo , L. Iannello , and I. B. Fekih , “Complementary Strategies for Biological Control of Aphids and Related Virus Transmission in Sugar Beet to Replace Neonicotinoids,” Agriculture 12, no. 10 (2022): 1663, 10.3390/agriculture12101663.

[39] O. M. Finkel , G. Castrillo , S. Herrera Paredes , I. Salas González , and J. L. Dangl , “Understanding and exploiting plant beneficial microbes,” Current Opinion in Plant Biology 38 (2017): 155–163, 10.1016/j.pbi.2017.04.018. 28622659 PMC5561662

[40] P. Pandey , A. Tripathi , S. Dwivedi , K. Lal , and T. Jhang , “Deciphering the mechanisms, hormonal signaling, and potential applications of endophytic microbes to mediate stress tolerance in medicinal plants,” Frontiers in Plant Science 14 (2023): 1250020, 10.3389/fpls.2023.1250020. 38034581 PMC10684941

[41] Y. Wang , S. Song , W. Zhang , et al., “Deciphering phenylalanine‐Derived Salicylic Acid Biosynthesis in Plants,” Nature 645, no. 8079 (2025): 208–217, 10.1038/s41586-025-09280-9. 40702180 PMC12408371

[42] J. Wu , W. Zhu , and Q. Zhao , “Salicylic Acid Biosynthesis is not from Phenylalanine in *Arabidopsis* ,” Journal of Integrative Plant Biology 65, no. 4 (2023): 881–887, 10.1111/jipb.13410. 36377737

[43] B. Manthe , M. Schulz , and H. Schnabl , “Effects of salicylic acid on growth and stomatal movements of *Vicia faba* L.: Evidence for salicylic acid metabolization,” Journal of Chemical Ecology 18, no. 9 (1992): 1525–1539, 10.1007/bf00993226. 24254284

[44] M. Berrocal‐Lobo and A. Molina , “Ethylene Response Factor 1 Mediates Arabidopsis Resistance to the Soilborne Fungus *Fusarium oxysporum* ,” Molecular Plant‐Microbe Interactions 17, no. 7 (2004): 763–770, 10.1094/mpmi.2004.17.7.763. 15242170

[45] P. Ding and Y. Ding , “Stories of Salicylic Acid: A Plant Defense Hormone,” Trends in Plant Science 25, no. 6 (2020): 549–565, 10.1016/j.tplants.2020.01.004. 32407695

[46] J. Ali , A. Tonğa , T. Islam , et al., “Defense strategies and associated phytohormonal regulation in *Brassica* plants in response to chewing and sap‐sucking insects,” Frontiers in Plant Science 15 (2024): 1376917, 10.3389/fpls.2024.1376917. 38645389 PMC11026728

[47] J. P. Métraux , H. Signer , J. Ryals , et al., “Increase in Salicylic Acid at the Onset of Systemic Acquired Resistance in Cucumber,” Science 250, no. 4983 (1990): 1004, 10.1126/science.250.4983.1004. 17746926

[48] A. Manikandan , R. Parthasarathy , S. Anusuya , and H. Jianying , “An Overview of Plant Defense‐related Enzymes Responses to Biotic Stresses,” Plant Gene 27, no. 4 (2021): 100302, 10.1016/j.plgene.2021.100302.

[49] K. W. Bock , “The UDP‐glycosyltransferase (UGT) superfamily expressed in humans, insects and plants: Animal‐plant arms‐race and co‐evolution,” Biochemical Pharmacology 99 (2016): 11–17, 10.1016/j.bcp.2015.10.001. 26453144

[50] D. Dimunová , P. Matoušková , R. Podlipná , I. Boušová , and L. Skálová , “The ROLe of UDP‐Glycosyltransferases in Xenobioticresistance,” Drug Metabolism Reviews 54, no. 3 (2022): 282, 10.1080/03602532.2022.2083632. 35635097

[51] Y. Pan , S. Wen , X. Chen , et al., “UDP‐glycosyltransferases contribute to spirotetramat resistance in *Aphis gossypii* Glover,” Pesticide Biochemistry and Physiology 166 (2020): 104565, 10.1016/j.pestbp.2020.104565. 32448419

[52] A. Pym , P. A. Umina , J. Reidy‐Crofts , et al., “Overexpression of UDP‐glucuronosyltransferase and cytochrome P450 enzymes confers resistance to sulfoxaflor in field populations of the aphid, *Myzus persicae* ,” Insect Biochemistry and Molecular Biology 143 (2022): 103743, 10.1016/j.ibmb.2022.103743. 35202811

[53] M. P. Donovan , P. D. Nabity , and E. H. DeLucia , “Salicylic acid‐mediated reductions in yield in Nicotiana attenuata challenged by aphid herbivory,” Arthropod‐Plant Interactions 7, no. 1 (2013): 45–52, 10.1007/s11829-012-9220-5.

