# Supplementary material for: Cotton Recruits Soil‐Derived Delftia tsuruhatensis to Suppress Aphid Detoxification Via Salicylic Acid‐Mediated Defense
Source: Adv Sci (Weinh). 2026 Apr 20;13(38):e75321. doi: 10.1002/advs.75321 (PMC13335700; doi:10.1002/advs.75321)
Supplement: Supplementary file 1 — Supporting File: advs75321‐sup‐0001‐SuppMat.docx. [file ADVS-13-e75321-s001.docx]

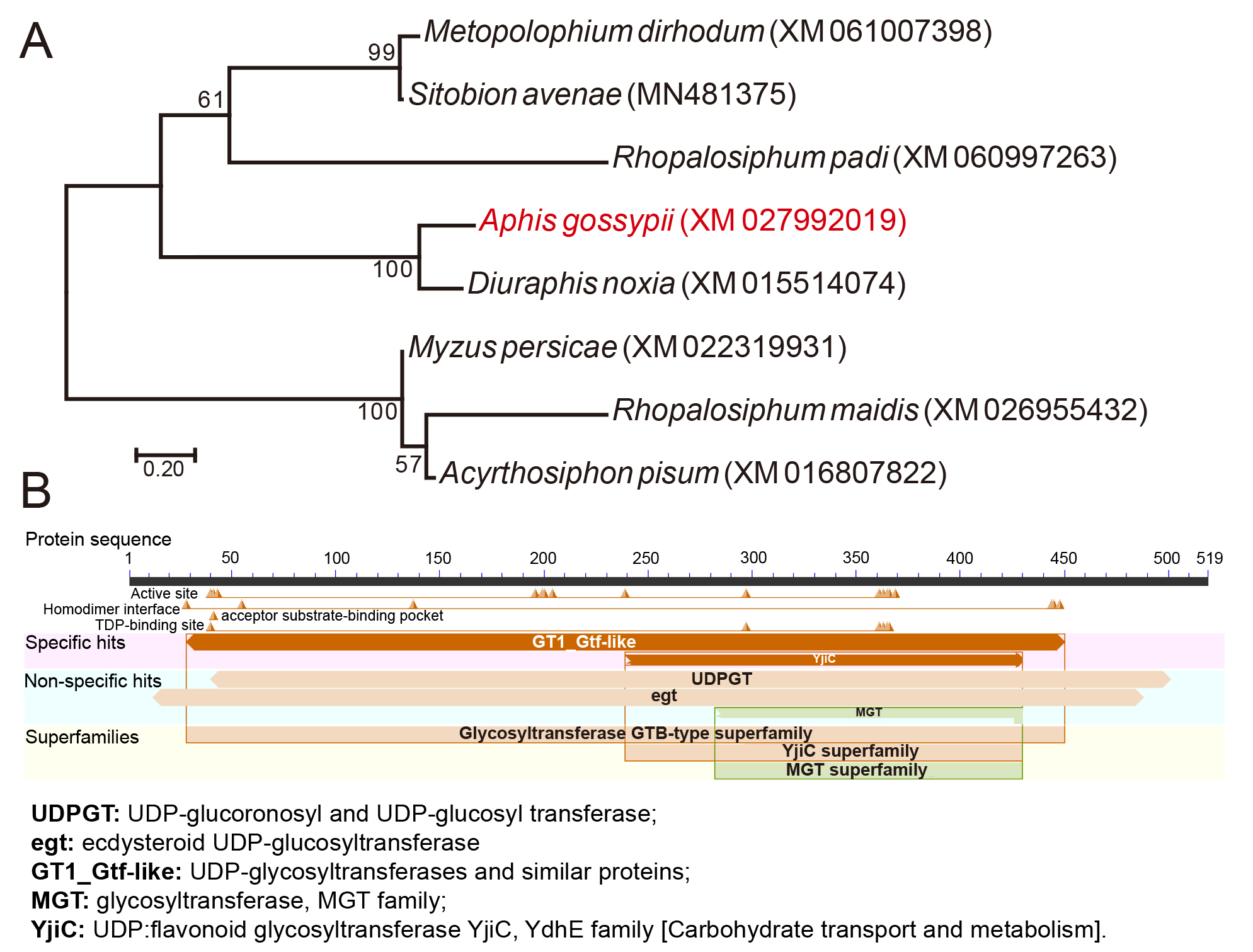


**Fig. S1** Bioinformatics analysis of *UGT2B17*. **(A)** Phylogenetic analysis of *UGT2B17*. Numbers in parentheses indicate NCBI accession numbers. **(B)** Predicted protein domains of *UGT2B17*.


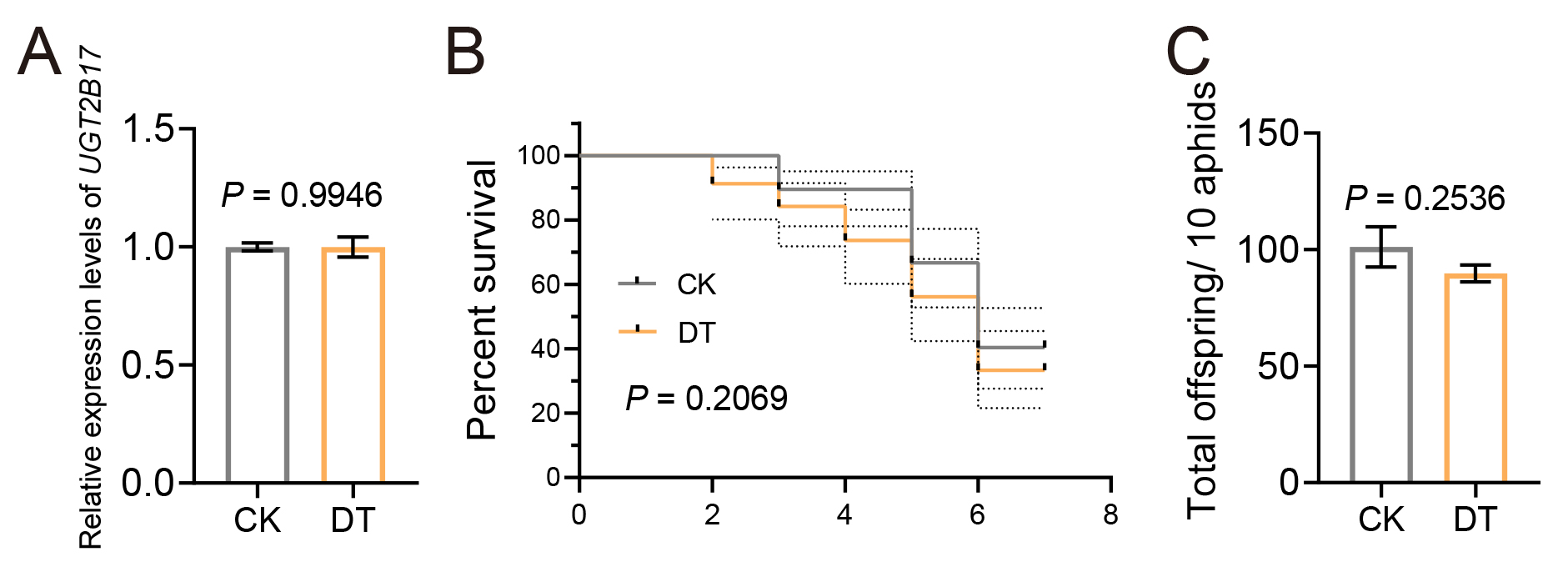


**Fig. S2** *D. tsuruhatensis* does not directly inhibit the fitness of cotton aphids. Direct feeding on *D. tsuruhatensis* did not result in significant changes in **(A)** *UGT2B17* expression, **(B)** survival rate, and **(C)** offspring number. Survival curves were compared using the log-rank (Mantel–Cox) test (*P* < 0.05). Pairwise comparisons were performed using Student’s *t*-test (*P* < 0.05).

| Number | Latin name | Separation site |
| --- | --- | --- |
| L-1 | *Agrococcus jenensis* | Cotton leaf |
| L-2 | *Alicyclobacillus sp* | Cotton leaf |
| L-3 | *Bacillus altitudinis* | Cotton leaf |
| L-4 | *Bacillus cereus* | Cotton leaf |
| L-5 | *Bacillus mobilicus* | Cotton leaf |
| L-6 | *Bacillus pacificus* | Cotton leaf |
| L-7 | *Bacillus pumilus* | Cotton leaf |
| L-8 | *Bacillus subtilis* | Cotton leaf |
| L-9 | *Bacillus thuringiensis* | Cotton leaf |
| L-10 | *Cronobacter dublinensis* | Cotton leaf |
| L-11 | *Delftia tsuruhatensis* | Cotton leaf |
| L-12 | *Moraxella osloensis* | Cotton leaf |
| L-13 | *Moraxellaceae bacterium* | Cotton leaf |
| L-14 | *Pantoea dispersa* | Cotton leaf |
| L-15 | *Peribacillus frigoritolerans* | Cotton leaf |
| L-16 | *Priestia megaterium* | Cotton leaf |
| L-17 | *Stenotrophomonas maltophilia* | Cotton leaf |
| L-18 | *Streptomyces rhizophilus* | Cotton leaf |
| L-19 | *Xanthomonas sp* | Cotton leaf |
| R-1 | *Achromobacter marplatensis* | Cotton root |
| R-2 | *Alicyclobacillus sp. LC-8* | Cotton root |
| R-3 | *Atlantibacter hermannii* | Cotton root |
| R-4 | *Bacillus licheniformis* | Cotton root |
| R-5 | *Bacillus paramycoides* | Cotton root |
| R-6 | *Bacillus thuringiensis* | Cotton root |
| R-7 | *Caulobacter flavus* | Cotton root |
| R-8 | *Chryseobacterium sp* | Cotton root |
| R-9 | *Dyella jiangningensis* | Cotton root |
| R-10 | *Ensifer adhaerens* | Cotton root |
| R-11 | *Enterobacter cloacae* | Cotton root |
| R-12 | *Enterobacter ludwigii* | Cotton root |
| R-13 | *Paenibacillus lautus* | Cotton root |
| R-14 | *Paenibacillus tundrae* | Cotton root |
| R-15 | *Paenibacillus xylanexedens* | Cotton root |
| R-16 | *Pseudoduganella violaceinigra* | Cotton root |
| R-17 | *Pseudomonas sp* | Cotton root |
| R-18 | *Pseudomonas plecoglossicida* | Cotton root |
| R-19 | *Pseudomonas putida* | Cotton root |
| R-20 | *Pseudomonas sp. BcB153* | Cotton root |
| R-21 | *Stenotrophomonas* | Cotton root |
| R-22 | *Xanthomonas sp. 33DCP* | Cotton root |

**Table S1** The culturable bacteria present in both the leaves and roots of cotton plants.

Note: L: Cotton leaves; R: Cotton root.

| **Primer name** | **Forward primer (5'-3')** | **Reverse primer (5'-3')** |
| --- | --- | --- |
| **Primers for detecting the copy number of *Delftia tsuruhatensis*** | | |
| qDT | TCACAAATGCAGTTCCCAGGT | GCGGTAATACGTAGGGTGCG |
| **Primers for *Delftia tsuruhatensis* clones** | | |
| pDT | CTGCCATGTCAAAGGTGGGT | GGTGCGAGCGTTAATCGGAA |
| **Primers for detecting the expression level of salicylic acid synthesis genes** | | |
| GhUBI-11 | CTGAATCTTCGCTTTCACGTTATC | GGGATGCAAATCTTCGTGAAAAC |
| qGhICS | GTCTTCAGCCACCTAATGGACCCGC | GCTCTGGATTCACCTCTAGCACG |
| qGhEDS5 | TCGGAGCTATCCTTGGACTG | CCCAACGTGAAGCATCCAAC |
| qGhPBS3 | ATTGCCAATGGGGAGCCATC | AGAGTTTCGGCGTTGACAGG |
| qGhEPS1 | AATCTCGGTTGGGCTGCTTG | GCATTGACGAAACCACGCAC |
| qGhPAL | AATGTCTGCCCAATTCTC | CTAGTGACCGGATTAGCAAG |
| qGhBEBT | TTGTTTATGGCGTTGTCGAACCATCG | ATGGGTTTTGACAAAGTTTCCCTGC |
| qGhBBH | CTCAATTGCCAAAGAGACTATGCG | GGTTATCCCAAACCGTGGGGTC |
| qGhBSE | CCATCCCATTACTTGTCCTATGGGG | AGGGTCAGTATCAATGGCAATCTTG |
| **Primers for** **the synthesis of ds*UGT2B17*** | | |
| T7UGT2B17 | TAATACGACTCACTATAGGG AAATGGTTGCCTCAACGTGA | TAATACGACTCACTATAGGG CCAATAAAAAGTATTGGTACCACG |
| **Primers for qRT-PCR of *UGT2B17*** | | |
| EF1α | GAAGCCTGGTATGGTTGTCGT | GGGTGGGTTGTTCTTTGTG |
| qUGT2B17 | TGCCAGTGATACACACGAGTC | TGTTGATACCGTTGCAGGGTT |

**Table S2** The primers used in this study
